# Supplementary material for: Novel Method for Early Prediction of Clinically Significant Drug–Drug Interactions with a Machine Learning Algorithm Based on Risk Matrix Analysis in the NICU
Source: J Clin Med. 2022 Aug 12;11(16):4715. doi: 10.3390/jcm11164715 (PMC9410171; doi:10.3390/jcm11164715)
Supplement: Supplementary file 1 [file jcm-11-04715-s001.zip › jcm-1853351-supplementary.pdf]

Table S1. Distribution of drugs used by patients included in the study (n = 2280).

Drugs	n	%	Drugs	n	%
1. Intravenous fluids [A]	275	12.06	67. Hydrochlorothiazide + spironolactone [C]	4	0.18
2. Gentamicin [J]	183	8.03	68. Terlipressin [H]	3	0.13
3. Ampicillin [J]	178	7.81	69. Amiodarone [C]	2	0.09
4. Vitamin K [B]	134	5.88	70. Azithromycin [J]	2	0.09
5. Multivitamin [A]	121	5.30	71. Calcium lactat [A]	2	0.09
6. Amikacin [J]	119	5.22	72. Zinc formulation [A]	2	0.09
7. Vankomycin [J]	115	5.04	73. Fibrinogen [B]	2	0.09
8. Meropenem [J]	112	4.91	74. Filgrastim [L]	2	0.09
9. Fluconazole [J]	111	4.87	75. Flecainide [C]	2	0.09
10. Dexmedetomidine [N]	59	2.59	76. Heparin [B]	2	0.09
11. Vitamin D [A]	49	2.15	77. Calcitriol [A]	2	0.09
12. Fentanyl [N]	46	2.02	78. Carnitine [A]	2	0.09
13. Caffeine [N]	43	1.89	79. Colistin [J]	2	0.09
14. Surfactant preparations [R]	43	1.89	80. Pancrelipase [H]	2	0.09
15. Probiotics [A]	30	1.32	81. Sodium benzoate [A]	2	0.09
16. Midazolam [N]	30	1.32	82. Netilmicin eyedrop [S]	2	0.09
17. Dexamethasone [H]	28	1.23	83. Nifedipine [C]	2	0.09
18. Dopamine [C]	27	1.18	84. Octreotide [H]	2	0.09
19. Furosemid [C]	26	1.14	85. Ornidazole [J]	2	0.09
20. Salbutamol [R]	26	1.14	86. Piperacillin-tazobactam [J]	2	0.09
21. Calcium Gluconate [A]	25	1.10	87. Sodium citrate-citric acid solution [A]	2	0.09
22. Potassium chloride [A]	25	1.10	88. Spironolactone [C]	2	0.09
23. Vitamin A [A]	23	1.01	89. Teicoplanine [J]	2	0.09
24. Phenobarbital [N]	22	0.96	90. Ophthalmic lubricant [S]	2	0.09
25. Cefazolin [J]	20	0.88	91. Topiramate [S]	2	0.09
26. Alprostadil [C]	19	0.83	92. Tropicamide eyedrop [S]	2	0.09
27. Hydrocortisone [H]	15	0.66	93. Ursodiol [A]	2	0.09
28. Milrinone [C]	15	0.66	94. Sodium bicarbonate [A]	2	0.08
29. Metronidazole [J]	13	0.57	95. Amlodipine [C]	1	0.04
30. Adrenaline [C]	12	0.53	96. Bleomycin [L]	1	0.04
31. Amoxicillin [J]	12	0.53	97. Dekas [A]	1	0.04
32. Enoxaparin [B]	12	0.53	98. Desmopressin [H]	1	0.04
33. Ciprofloxacin [J]	12	0.53	99. Diazepam [N]	1	0.04
34. Allopurinol [M]	11	0.48	100. Dorzolamide eyedrop [S]	1	0.04
35. Sodium chloride [A]	11	0.48	101. Etopozid [L]	1	0.04
36. Tobramycin [S]	11	0.48	102. Phenylephrine [S]	1	0.04
37. Ferrous fumarate [B]	10	0.44	103. Enema [S]	1	0.04
38. Levetiracetam [N]	10	0.44	104. Fludarabine [L]	1	0.04
39. Ibuprofen [C]	9	0.39	105. Aluminum hydroxide [A]	1	0.04
40. Morphine [N]	9	0.39	106. Glucagon [H]	1	0.04
41. Propranolol [C]	9	0.39	107. Iloprost [B]	1	0.04
42. Diazoxide [C]	8	0.35	108. Imipenem [J]	1	0.04
43. Levothyroxine [H]	8	0.35	109. Immune globulin [B]	1	0.04
44. Sildenafil [G]	8	0.35	110. Cabergoline [G]	1	0.04
45. Adenosine[C]	7	0.31	111. Potassium citrate [A]	1	0.04
46. Hydrochlorothiazide [C]	7	0.31	112. Activated charcoal [A]	1	0.04
47. Captopril [C]	7	0.31	113. Carboplatin [L]	1	0.04
48. Acetylcysteine [R]	7	0.31	114. Ketamine [N]	1	0.04
49. Acetylsalicylic acid [B]	6	0.26	115. Latanoprost eyedrop [S]	1	0.04
50. Albumin [B]	5	0.22	116. Medroxyprogesterone acetate [G]	1	0.04
51. Dornase alpha [R]	5	0.22	117. Magnessium sulphate [A]	1	0.04
52. İnsulin [H]	5	0.22	118. Mesenchymal stem cell [B]	1	0.04
53. Penicillin G [J]	5	0.22	119. Mupirocin [S]	1	0.04

J

54.	Dexamethasone eyedrop [S]	4	0.18	120.	Nitroglycerine [C]	1	0.04
55.	Phenytoin [N]	4	0.18	121.	Oseltamivir [J]	1	0.04
56.	Linezolid [J]	4	0.18	122.	Pantoprazole [A]	1	0.04
57.	Methylprednisolone [H]	4	0.18	123.	Prednisolone [H]	1	0.04
58.	Paracetamol [N]	4	0.18	124.	Pyridoxine [A]	1	0.04
59.	Ranitidine [A]	4	0.18	125.	Ceftriaxone [J]	1	0.04
60.	Ampicillin + sulbactam [J]	4	0.18	126.	Cefuroxime [J]	1	0.04
61.	Biotin [A]	3	0.13	127.	Sotalol [C]	1	0.04
62.	Levosimendan [C]	3	0.13	128.	Tranexamic acid [B]	1	0.04
63.	Metylene blue [C]	3	0.13	129.	Vinblastine [L]	1	0.04
64.	Nitrofurazone [S]	3	0.13	130.	Vitamin B [A]	1	0.04
65.	Noradrenaline [C]	3	0.13	131.	Vitamin C [A]	1	0.04
66.	Cefotaxime [J]	3	0.13		Total	2280	100

[A.] Alimentary tract and metabolism, [B.] Blood and blood-forming organs, [C.] Cardiovascular system, [G.] Genito-urinary system and sex hormones, [H.] Systemic hormonal preparations, [J.] Anti-infectives for systemic use, [L.] Antineoplastic and immunomodulating agents, [M.] Musculo-skeletal system, [N.] Nervous system, [R.] Respiratory system, [S.] Sensory organs.
